# Influence of Oxygen Pressure on the Domain Dynamics and Local Electrical Properties of BiFe_0.95_Mn_0.05_O_3_ Thin Films Studied by Piezoresponse Force Microscopy and Conductive Atomic Force Microscopy

**DOI:** 10.3390/ma10111258

**Published:** 2017-11-01

**Authors:** Kunyu Zhao, Huizhu Yu, Jian Zou, Huarong Zeng, Guorong Li, Xiaomin Li

**Affiliations:** 1 Key Laboratory of Inorganic Functional Materials and Devices, Shanghai Institute of Ceramics, Chinese Academy of Sciences, Shanghai 200050, China; zhaokunyu@mail.sic.ac.cn (K.Z.); jianzou@student.sic.ac.cn (J.Z.); grli@mail.sic.ac.cn (G.L.); 2 School of Chemistry and Materials Engineering, Fuyang Normal University, Fuyang 236037, China; yuhuizhu1017@163.com; 3 University of Chinese Academy of Sciences, Beijing 100039, China; 4 State Key Laboratory of High Performance Ceramics and Superfine Microstructures, Shanghai Institute of Ceramics, Chinese Academy of Sciences, Shanghai 200050, China; lixm@mail.sic.ac.cn

**Keywords:** multiferroic BiFe_0.95_Mn_0.05_O_3_ film, PFM, conductive-AFM, domain structure

## Abstract

In this work, we have studied the microstructures, nanodomains, polarization preservation behaviors, and electrical properties of BiFe_0.95_Mn_0.05_O_3_ (BFMO) multiferroic thin films, which have been epitaxially created on the substrates of SrRuO_3_, SrTiO_3_, and TiN-buffered (001)-oriented Si at different oxygen pressures via piezoresponse force microscopy and conductive atomic force microscopy. We found that the pure phase state, inhomogeneous piezoresponse force microscopy (PFM) response, low leakage current with unidirectional diode-like properties, and orientation-dependent polarization reversal properties were found in BFMO thin films deposited at low oxygen pressure. Meanwhile, these films under high oxygen pressures resulted in impurities in the secondary phase in BFMO films, which caused a greater leakage that hindered the polarization preservation capability. Thus, this shows the important impact of the oxygen pressure on modulating the physical effects of BFMO films.

## 1. Introduction

In recent years, multiferroic materials have attracted renewed research interest due to their electric, magnetic, and structural order parameters, which might give rise to next generation electronic devices. These properties facilitate the magnetic (spin) state control by electric fields, with some having the potential to be used in multistate logic sensors, non-volatile memories, solid-state transformers, and electromagneto-optic actuators [1,2,3,4,5,6]. Among multiferroic materials, BiFeO_3_ (BFO) has been intensively investigated due to its unique characteristics of a high ferroelectric–paraelectric transition temperature (*T_C_*) of approximately 1083 K, and high antiferromagnetic to paramagnetic Néel temperature (*T_N_*) of approximately 625 K [7,8,9,10]. However, it is well known that BFO causes high leakage currents and magnetic measurement errors that have been attributed to specific structural defects [11,12,13,14]. Heretofore, many studies concentrate on decreasing leakage properties via the doping method, especially using a partial substitution of Fe element for Mn [15,16,17,18,19,20,21]. However, little effort has been taken to understand the impact of oxygen pressure on the nanoscale microstructures, domain dynamics, and resistive properties.

As common and powerful tools, piezoresponse force microscopy (PFM) and conductive atomic force microscopy (C-AFM) have been extensively adopted for investigating nanoscale ferroelectrics [22,23,24]. Compared with the other classical domain characterization techniques, PFM has distinct advantages with respect to its super-high resolution and local electrical poling ability based on tip-bias, which make it a powerful method for investigating domain structures at the nanoscale. In this paper, we used piezoresponse force microscopy and conductive atomic force microscopy to study the local electrical properties and leakage current of Mn-doped BFO films prepared by the pulsed laser deposition (PLD) method under different oxygen pressure deposition conditions. It can be found that oxygen pressure plays a key role in the nanoscale domain structures and the related electrical properties.

## 2. Materials and Methods

In order to compensate the volatilization of Bismuth during the film deposition process, Bi_1.15_Fe_0.9_5Mn_0.05_O_3_ (BFMO) (15% Bi-rich) ceramic is chosen as the target. The pulsed laser deposition (PLD) method was used to prepare 200 nm-thick BFMO films. By using a KrF exciter laser (248 nm, Lamda Phsik COM PexPro 201 (Lambda Physik, Fort Lauderdale, FL, USA)) with an energy density of 7 J/cm^2^ and 5-Hz frequency, the deposition of the BFMO thin film was performed at 570 °C, with oxygen pressure fixed at 2 Pa and 10 Pa. Buffer layers composed of epitaxial SrRuO_3_ (SRO) (50 nm), SrTiO_3_ (STO) (25 nm), and TiN (10 nm) films were grown on HF-cleaned Si(001) substrates by laser molecular beam epitaxy before the preparation of BFMO films. In our previous studies, only (001) reflections were revealed, confirming the purity and epitaxial growth of the films [15]. 

The ferroelectric domain structures were studied via the piezoresponse force microscopy (PFM), which was developed based on a commercial scanning probe microscope (SPA 400, Seiko Inc., Tokyo, Japan). A lock-in amplifier (Signal Recovery 7208 (Zurich Instruments, Zürich, Switzerland)) and a function generator (Agilent 3301 (Erecycler LLC, Dallas, TX, USA)) were used as equipment for the PFM. The working principle of the piezoresponse force microscopy is related to the strength of the examination of the converse piezoelectric effect, which creates a local piezoelectric vibration signal due to an external AC voltage applied between the conductive probe and the bottom electrode of the sample. The magnitude of the effective piezoelectric coefficient and the polarization orientation of the sample have a linear relationship with the amplitude and phase PFM signal, and can be used to determine these values. In PFM, we used a silicon cantilever coated by platinum/titanium, whose spring constants and resonance frequency are 2 N/m and 70 kHz, respectively. C-AFM (AFM/Current Imaging Tunneling Spectroscopy (AFM-CITS) mode (Hitachi High-Technologies Corporation, Tokyo, Japan)), with the same tip as PFM, was utilized to perform local current–voltage (I–V) property measurements. All experiments were conducted in an ambient environment.

## 3. Results and Discussion

**Figure 1:**
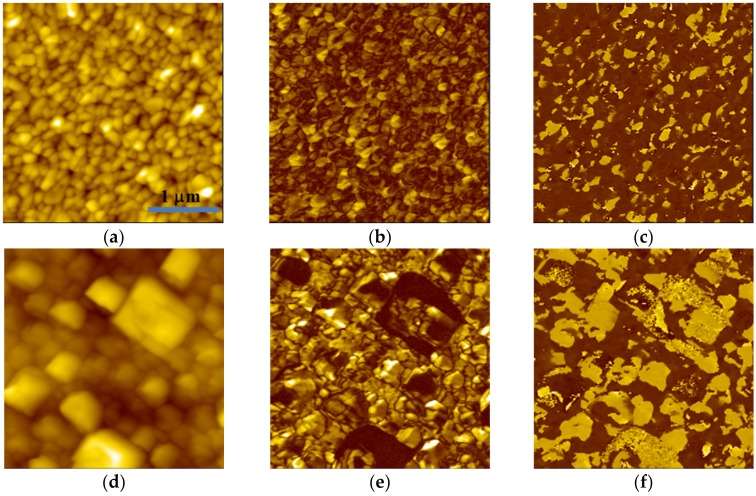
Topography images of BiFe_0.95_Mn_0.05_O_3_ (BFMO) films with the deposition at oxygen pressures of (**a**) 2 Pa and (**d**) 10 Pa respectively, with the (**b**,**e**) corresponding piezoresponse force microscopy (PFM) amplitude images and the (**c**,**f**) phase image.

**Figure 2:**
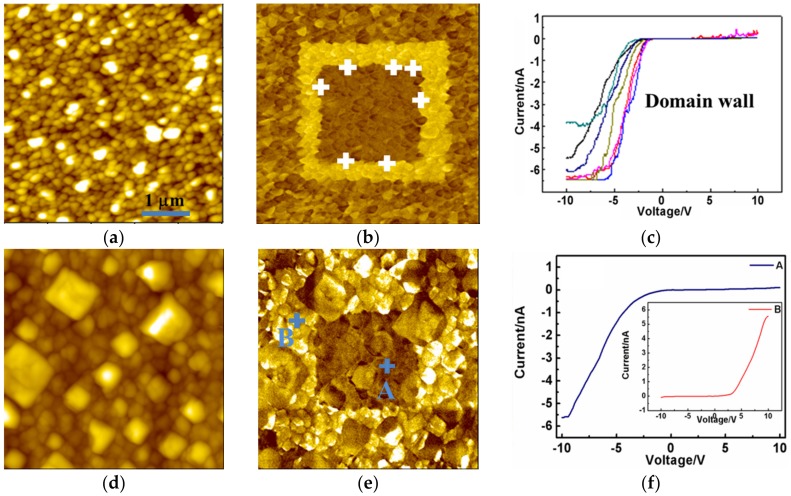
Topography images of BiFe_0.95_Mn_0.05_O_3_ (BFMO) films with the deposition at oxygen pressures of (**a**) 2 Pa and (**d**) 10 Pa respectively, with the (**b**,**e**) corresponding piezoresponse force microscopy (PFM) phase images and the (**c**,**f**) I–V curves of BFMO films.

BFMO thin films preferentially grow in the SrRuO_3_ (SRO) orientation. A higher oxygen pressure would impair the growth and combination of thin films induced by the substrate under the same temperature, which leads to the formation of the polycrystalline state in BFMO. BFMO films deposited at an oxygen pressure of 2 Pa possess a single phase, while BFMO obtained at P_O₂_ = 10 Pa contains a Bi_2_O_3_ phase [15]. Figure 1 exhibits the topography (Figure 1a,d), as well as the corresponding PFM amplitude image (Figure 1b,e) and PFM phase image (Figure 1c,f) of BFMO obtained at P_O₂_ of 2 Pa and 10 Pa, respectively. A homogeneous grain size can be observed in the BFMO film prepared at a P_O₂_ of 2 Pa, with approximately 100 nm width (see Figure 2a). In contrast, the grain size of the BFMO film at a P_O₂_ of 10 Pa is inhomogeneous, with an average width of 300 nm. This is obviously larger than those of BFMO films deposited at an oxygen pressure of 2 Pa, as shown by Figure 1d. The formation of square-shaped grains is attributed to the overgrowth of the Bi_2_O_3_ phase, which leads to the appearance of self-organized arrays [25]. In the surface of BFMO thin films, the abnormal growth of grains and the Bi_2_O_3_ phase occurred under high oxygen pressures. These results are consistent with X-ray diffraction (XRD) results in a previous study [15]. In PFM images, single domain or multi-domain states can be observed in the individual grain. We found that the PFM amplitude image exhibits a bright, dark, and gray tone for piezoresponse contrasts. The PFM amplitude signal is closely related to the local piezoelectric coefficient. Based on the PFM imaging principle, a higher piezoelectricity results in a greater contrast in the piezoresponse amplitude image. Thus, in BFMO thin films, grains display notably inhomogeneous piezoresponse characteristics, which are attributed to individual stress impacts around the grains [26]. Random distributional ferroelectric domains exist in both Mn-doped BFO films at oxygen pressures of 2 Pa and 10 Pa, which is observed by out-of-plane and in-plane phase images (Figure S1). Null piezoresponse contrasts can be observed in the PFM amplitude image (Figure 1e), which correspond to the Bi_2_O_3_impurity phase. It can be deduced that the oxygen pressure conditions applied during the BFMO thin film deposition process have a significant influence on the microstructures and nanoscale domain features.

To obtain deeper insight into the impact of oxygen pressure on the polarization switching performances, the PFM tip with a direct current (DC) bias was adopted to create a domain with opposite polarization orientation. Firstly, one 3 µm × 3 µm region was polarized with the −5 V DC bias in the initial 5 µm × 5 µm region, before the central 2 µm × 2 µm region was polarized with a 5 V tip bias. The PFM results are shown in Figure 2. BFMO deposited at a P_O₂_ of 2 Pa exhibit a highly contrasted PFM phase image, due to its low leakage current. Furthermore, an anti-parallel polarization orientation was revealed in the polarized domain (Figure 2b). Figure 2e shows a relatively obscure piezoresponse contrast in the BFMO at a high oxygen pressure, which is possible due to the poor polarization state generated by high leakage properties. 

Local current–voltage (I–V) behaviors of domain boundaries in BFMO thin films were investigated via C-AFM in order to understand the effect of oxygen pressure at the nanoscale leakage behavior of BFMO films. Figure 2c shows the I–V curves near the domain boundaries in BFMO films at a P_O₂_ of 2 Pa. It was found that the conductive properties of thin films show an obvious discrepancy when bias is applied at different orientations. When a negative bias voltage was applied, the local current was almost invariable under the low voltage stage, and increased remarkably beyond a threshold voltage, exhibiting a conductive state. When a positive bias voltage was applied, the local current maintained a low level, and was stable under different bias voltages, revealing a cut-off state. These unidirectional conductive performances in BFMO films at a P_O₂_ of 2 Pa revealed by I–V measurements imply that there might be some potential barrier, which may hinder the motion of the charge carrier towards a certain orientation. Asymmetry bending may occur in the energy band structure at the domain wall, which would lead to an asymmetry of conductive properties. In perovskite BiFeO_3_ thin films, oxygen vacancy acts as a donor electron, making BiFeO_3_ n-type semiconductors. The metal–semiconductor contact between tip and sample presents a rectifying effect that corresponds to Schottky contact. Thus, it can be deduced that the work function of metal Pt is larger than that of n-type semiconductor thin films. When contact between Pt and an n-type semiconductor appears under thermal equilibrium conditions, electrons would flow from the conduction band in BFO to Pt, forming a depletion layer with an upswept energy band in BFMO films, namely, a Schottky barrier.

Figure 2f shows the I–V curves of two points (point A and point B) from anti-parallel polarization orientation regions in Figure 2e in BFMO films at a P_O₂_ of 10 Pa. The C-AFM result of point A shows similar conductive performance with that in BFMO films at a P_O₂_ of 2 Pa. The I–V behavior of point B exhibits an inverse relationship, in which the current remained in the cut-off state under the negative bias voltage and showed unidirectional conductivity under positive bias. Similar I–V properties can be observed in BFMO films at a P_O₂_ of 2 Pa (Figure S2). For the Pt/BFO/SRO structure, it is noted that the Schottky-to-Ohmic interface contact between the metal electrode and BFMO films is closely linked to the polarization orientation. Oxygen vacancy acts as a donor electron, since BFMO belongs to the n-type semiconductor. Thus, the Schottky barrier or depletion layer in the Pt/BFMO interface could be modulated by the polarization charge. The neutralization of the positive polarization charge by electrons from oxygen vacancy will lead to the aggregation of electrons on one end of polarization, and bend the band downwards to form quasi-ohmic contact. Although the negative polarization charge can be compensated by oxygen vacancy with positive charges, there will finally be Schottky contact on the other end of polarization. Thus, the polarization reversal can be utilized to modulate the reversal of the ferroelectric diode.

**Figure 3:**
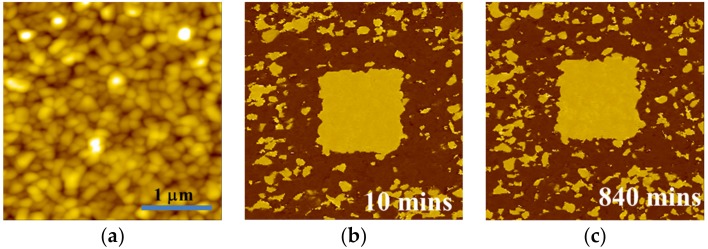
The (**a**) topography image of BFMO film with the deposition oxygen pressure of 2 Pa. The corresponding piezoresponse phase images are shown after being depolarized with −5 V voltage with the tip earthed for (**b**) 10 min and (**c**) 840 min.

**Figure 4:**
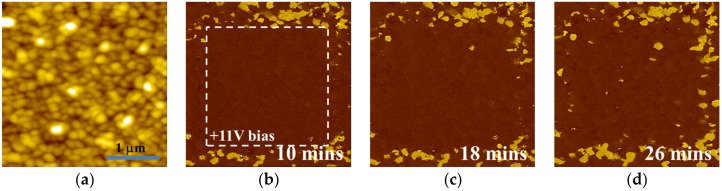

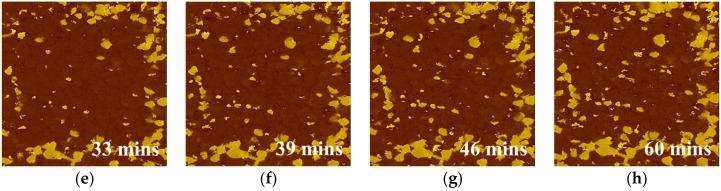
The (**a**) topography image of BFMO film with the deposition oxygen pressure of 2 Pa; (**b**–**h**) The corresponding out-of-plane piezoresponse phase images after depolarized with +11 V voltage with the tip earthed for different periods of time.

**Figure 5:**
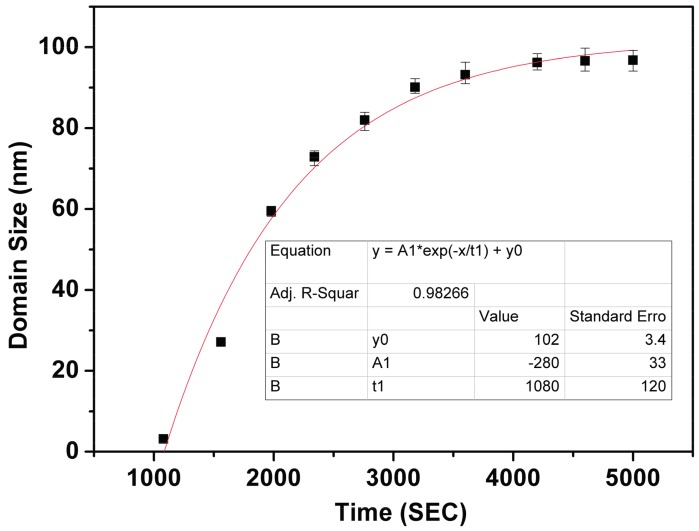
The relationship of domain size with time increasing in the poled area of Figure 4.

BFMO films at higher oxygen pressures (10 Pa) contain a conductive Bi_2_O_3_ phase, which leads to a high leakage current density in the sample. Thus, the preservation ability of polarization relaxation is hardly observed in BFMO at a P_O₂_ of 10 Pa. Therefore, the PFM results of the polarization relaxation properties in BFMO films at a P_O₂_ of 2 Pa is studied, with the results shown in Figure 3. First, the sample was polarized by −5 V bias voltage, with the tip earthed in 1 µm × 1 µm region, before the PFM measurement was performed in the 3 µm × 3 µm region. Figure 3b,c shows the PFM phase images of different time intervals after poling, which reveal a single domain state with downward polarization orientation after poling. Figure 3c shows that the polarization preservation remained stable after 24 h. However, BFMO films polarized with a positive tip bias exhibit remarkably different preservation behavior. In the initial 3 µm × 3 µm region, one 2 µm × 2 µm region was polarized with the +11 V bias with the tip earthed, before the PFM measurement was performed in the 3 µm × 3 µm region. Figure 4 gives piezoresponse phase images after different time intervals, with the relaxation times being 10 min, 18 min, 26 min, 33 min, 39 min, 46 min, and 60 min, respectively. It was found that under initial conditions, the poling area shows a completely single domain state, and an upward polarization orientation (Figure 4b). When the relaxation time increased, speckle-like domains appeared, which have an anti-parallel polarization orientation. These domains grow rapidly during the initial stage, and tend to be stable after 60 min. The relationship between the average size of all backswitched domains in the poled area in Figure 4 and relaxation time is shown in Figure 5, with the fitting function expressed as:\

D=280exp(t1080)+102 t ≥ 600  s

where *D* is domain radius, and *t* is relaxation time. It should be noted that for time below 600 s, since the domain backswitching cannot be observed in the PFM results, the time boundary condition is increased after the equation. The fitting curve may reflect the nucleation, growing up, and coalescing stage of domain growth process in the poled area. 

From the above results, it is seen that the polarization relaxation properties of BFMO films are related to the polarity of domains, which could be derived from the internal bias field caused by the composite defect in the sample. For pure BFO films, the volatilization of Bi and variable valent electrons of Fe^3+^ would give rise to a $V_{O}^{••}$−${\mathrm{Fe}}_{\mathrm{Fe}}^{\prime }$ composite defect. Thus, the Mn element will further induce a $V_{O}^{••}$−$A_{\mathrm{Fe}}^{\prime }$ (A: Fe, Mn) composite defect, by which the induced internal bias field is parallel to the polarization orientation (upward), and will enhance the stability of domains towards the same orientation. Domains with an anti-parallel polarization orientation can easily be switched back by the internal bias field. Thus, such an internal bias field derived from the composite defect will support the stability of the polarization in the same orientation, and hamper the polarization preservation in the reverse orientation. Evidently, that will be to the disadvantage of the application of BFO-based storage devices. Therefore, it is necessary to prepare high-performance BFO films with fewer defects by optimized technology. On the other hand, it is also beneficial to explore novel applications derived from composite defect-induced polarization fields by defect engineering in BFO materials.

## 4. Conclusions

In this study, the effects of oxygen pressure on the microstructures, domain features, local polarization preservation, and electrical properties of BFMO multiferroic thin films epitaxially grown on the substrates of SrRuO_3_, SrTiO_3_, and TiN-buffered (001)-oriented Si were investigated via piezoresponse force microscopy and conductive atomic force microscopy. It is found that BFMO films deposited at low oxygen pressures demonstrated a pure phase state, local electrical inhomogeneity, relatively low leakage with unidirectional diode-like performance, and orientation-dependent polarization reversal properties. In contrast, high oxygen pressures resulted in the Bi_2_O_3_ impurity phase in BFMO thin films, in which a higher leakage current prevents the polarization preservation behavior. This reveals the importance of modulating the physical properties in BFMO films by controlling oxygen pressures.

## Supplementary Materials

The following are available online at www.mdpi.com/1996-1944/10/11/1258/s1, Figure S1: The topography image (a,d) of BFMO films with the deposition oxygen pressures of 2 Pa and 10 Pa respectively, the corresponding out-of-plane PFM images (b,e) and the in-plane images (c,f),Figure S2: The I–V curves (c,f) of BFMO films deposited at a P_O_2__ of 2 Pa. The insert shows the position of points A, B, C and D, Figure S3: The topography image (a) of BFMO film with the deposition oxygen pressure of 2 Pa. The corresponding in-plane piezoresponse phase image (b–h) after being depolarized with +11 V voltage with the tip earthed for different periods of time, Table S1: The average size of all backswitched domains in the poled area in Figure 4.
