# Supplementary material for: Influence of Oxygen Pressure on the Domain Dynamics and Local Electrical Properties of BiFe0.95Mn0.05O3 Thin Films Studied by Piezoresponse Force Microscopy and Conductive Atomic Force Microscopy
Source: Materials (Basel). 2017 Nov 1;10(11):1258. doi: 10.3390/ma10111258 (PMC5706205; doi:10.3390/ma10111258)
Supplement: Supplementary file 1 [file materials-10-01258-s001.pdf]

Supplementary Materials

Influence of Oxygen Pressure on the Domain Dynamics and Local Electrical Properties of $BiFe_{0.95}Mn_{0.05}O_3$ Thin Films Studied by Piezoresponse Force Microscopy and Conductive Atomic Force Microscopy

Kunyu Zhao [1,†], Huizhu Yu [2,†], Jian Zou [1,3], Huarong Zeng [1,*], Guorong Li [1] and Xiaomin Li [4]

1 Key Laboratory of Inorganic Functional Materials and Devices, Shanghai Institute of Ceramics, Chinese Academy of Sciences, Shanghai 200050, China; zhaokunyu@mail.sic.ac.cn (K.Z.); jianzou@student.sic.ac.cn (J.Z.); huarongzeng@mail.sic.ac.cn (H.Z.); grli@mail.sic.ac.cn (G.L.)

2 School of Chemistry and Materials Engineering, Fuyang Normal University, Fuyang, Anhui 236037, China; yuhuizhu1017@163.com

3 University of Chinese Academy of Sciences, Beijing 100039, China

4 State Key Laboratory of High Performance Ceramics and Superfine Microstructures, Shanghai Institute of Ceramics, Chinese Academy of Sciences, Shanghai 200050, China; lixm@mail.sic.ac.cn

* Correspondence: huarongzeng@mail.sic.ac.cn; Tel.: +86-021-5241-1076

† Kunyu Zhao and Huizhu Yu contributed equally to this work.

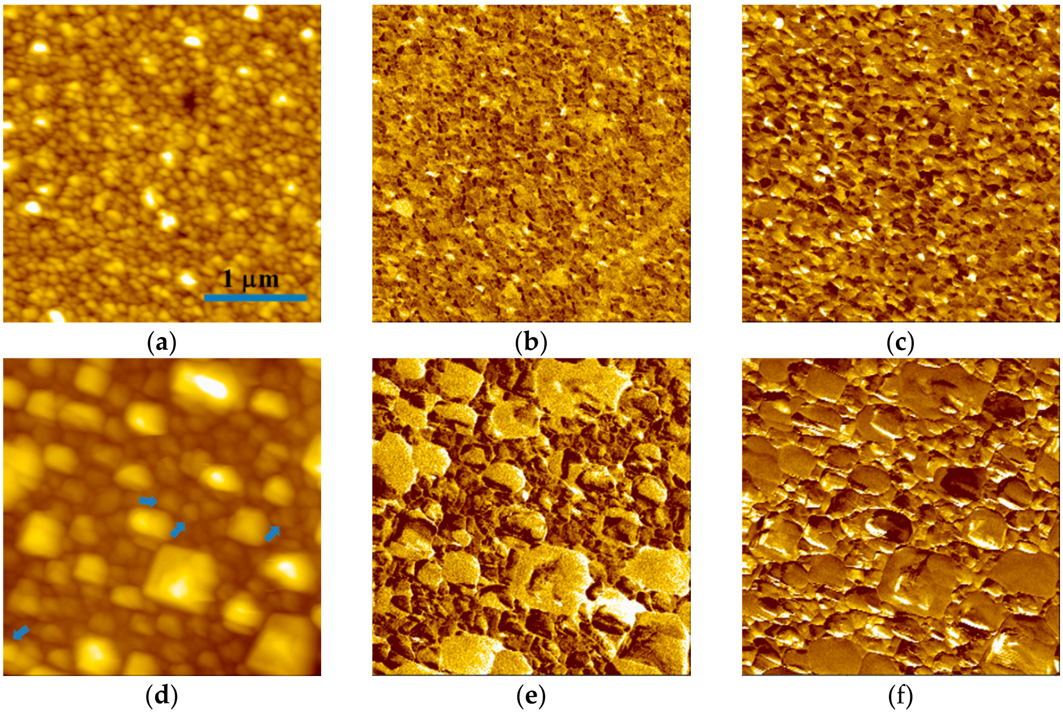

Figure S1. The topography image (**a**,**d**) of BFMO films with the deposition oxygen pressure 2 Pa and 10 Pa respectively, the corresponding out-of-plane PFM image (**b**,**e**) and the in-plane image (**c**,**f**).

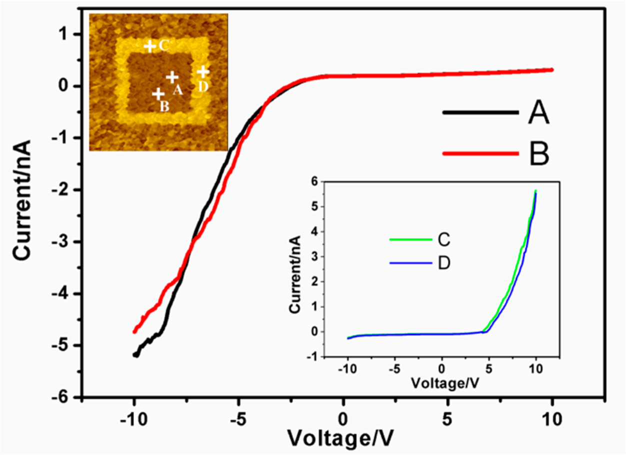

Figure S2. The I–V curves (c, f) of BFMO films deposited at P_{O_2} = 2 Pa. The insert shows the position of point A, B, C, and D.

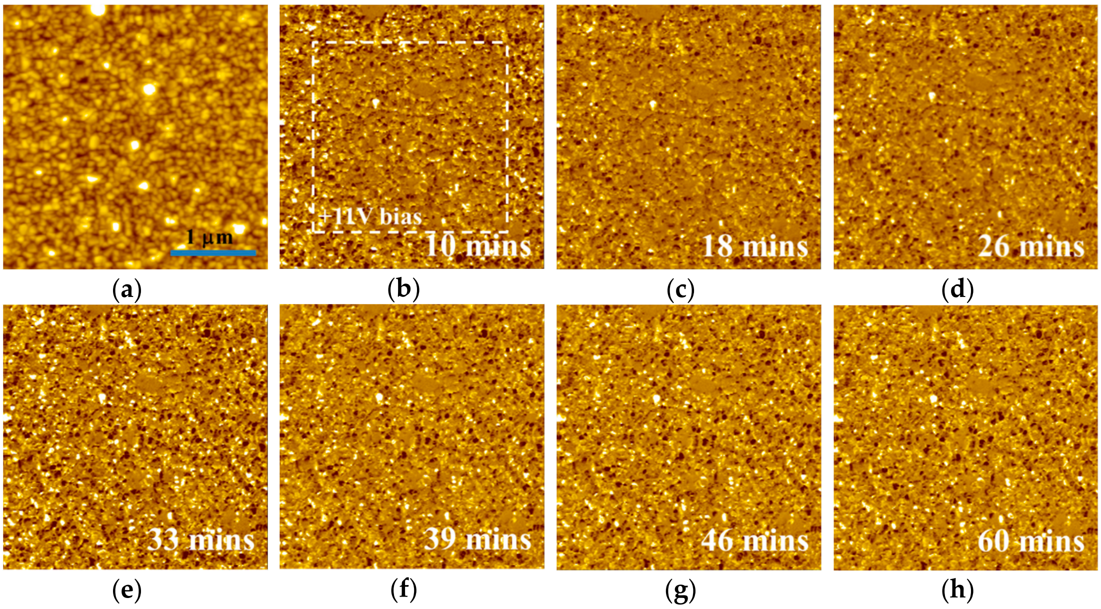

Figure S3. The topography image (**a**) of BFMO film with the deposition oxygen pressure P_{O_2} = 2 Pa. The corresponding in-plane piezoresponse phase image (**b–h**) after depolarized with +11 V voltage with the tip earthed at different times.

Table S1. The average size of all backswitched domains in the poled area in Figure 4.

Time (sec)	Average Domain Size (nm)
1080	3.2
1560	27.1
1980	59.5
2340	72.9
2760	82.0
3180	90.1
3600	93.2
4200	96.2
4600	96.6
5000	96.8
